# Editorial: Community series in novel biomarkers in tumor immunity and immunotherapy, volume II

**DOI:** 10.3389/fimmu.2025.1608903

**Published:** 2025-04-25

**Authors:** Eyad Elkord, Takaji Matsutani

**Affiliations:** ^1^ Department of Biosciences and Bioinformatics & Suzhou Municipal Key Lab of Biomedical Sciences and Translational Immunology, School of Science, Xi’an Jiaotong-Liverpool University, Suzhou, China; ^2^ College of Health Sciences, Abu Dhabi University, Abu Dhabi, United Arab Emirates; ^3^ Biomedical Research Center, School of Science, Engineering and Environment, University of Salford, Manchester, United Kingdom; ^4^ Translational Research Department, Drug Development Laboratories, Kyoto R&D Center, Maruho Co., Ltd., Kyoto, Japan; ^5^ Division of Medical Oncology/Hematology, Department of Medicine, Kobe University Graduate School of Medicine, Kobe, Japan

**Keywords:** biomarker, ICI, bioinformatics, tumor immunology, cancer

Cancer immunotherapy has recently revolutionized cancer treatment. However, response rates are still modest and further improvements are urgently needed. The identification of predictive biomarkers is critical for guiding treatment and predicting outcomes. This editorial highlights contributions from recent research, showcasing a range of innovative studies that explore biomarkers in tumor immunity and their implications for cancer immunotherapy.

This Research Topic comprises twelve original articles and one systematic review. The first article by Liu et al. identified a novel immune-based subtype of poor prognosis in breast cancer, which offers implications for immunotherapy targeting tertiary lymphoid structures. The study showed the critical roles of IL-6 and IL-6R, in addition to CD46 and JAG1, in the survival and invasive capacity of cancer cells, highlighting their potential as targets in antitumor therapies.

With the emergence of COVID-19 pandemic, the relationship between viral infections and cancer outcomes has attracted investigational interests. The second study by Wang et al. developed a SARS-CoV-2-related gene signature, which can be implemented to predict prognosis and mirror the immune landscape in the tumor microenvironment in patients with lung adenocarcinoma.

Single-cell RNA sequencing has transformed our understanding of the tumor microenvironment and tumor immunity. Using single-cell RNA-seq data from the GEO database, Fan et al. uncovered the role of T cell exhaustion in osteosarcoma. Three T-cell exhaustion-related genes (RAD23A, SAC3D1, PSIP1) were identified and used to formulate a T-cell exhaustion model. This data should be useful in designing interventional studies to reinvigorate the exhausted immune responses in osteosarcoma.

Predictive models for cancer therapies are an important area of research focus. The study by Shi et al. reported that PD-L1, tumor mutational burden (TMB) and neutrophils were prognostic biomarkers for a short-term efficacy of anti-PD-1 therapy combined with chemotherapy in patients with non-small cell lung cancer.

Immune-related adverse events (irAEs) associated with cancer immunotherapies are a concern. Xing et al. identified potential biomarkers for prediction of thyroid irAEs in patients with advanced gastrointestinal cancer. The study reported that elevated levels of adenosine deaminase (ADA) were associated with the occurrence of thyroid irAEs in these patients receiving anti-PD-1 therapy.

The immune system plays critical roles in the development and treatment of different cancers. Fang et al. have further dissected the role of the immune system in thyroid cancer. The study explored the genetic means for the involvement of immune cells in tumorigenesis, which may offer insights for future clinical research in thyroid cancer.

Inflammation plays key roles in the development and prognosis of cancers. A meta-analysis study by Wang et al. identified the prognostic value of systemic immune-inflammation index (SII) in osteosarcoma. The study reported that higher SII was significantly associated with poor overall survival and advanced stage.

The identification of telomere-related long non-coding RNAs (lncRNAs) is an area of interest in cancer. The study by Xu et al. established a prognostic model for the telomere-related lncRNAs (TRLs). A six-TRLs prognostic model has been identified in ovarian cancer, which offers an avenue for targeted therapies.

Artificial intelligence in the diagnosis and prognosis of cancer is gaining great interest. Using whole slide image-based weakly supervised deep learning, Han et al. developed models to predict major pathological response to neoadjuvant chemoimmunotherapy in non-small cell lung cancer patients.

Pain is a serious issue associated with cancer. The study by Zhang et al. investigating the correlation and potential mechanisms between pain and the efficacy of cancer immunotherapy. Interestingly, baseline pain was found to be an independent prognostic factor affecting the efficacy of immune checkpoint inhibitors in non-small cell lung cancer patients, potentially through promotion of CXCL12-mediated inflammation and immunosuppression.

In search for biomarkers for therapeutic response to immune checkpoint inhibitors, a nationwide multi-centric prospective study aimed to identify pretreatment plasma exosome mRNAs as predictive biomarkers for nivolumab, a PD-1 inhibitor in head and neck cancer patients. Sato et al. reported that the plasma exosome mRNAs signature may better predict which patients respond to nivolumab, ensuring a more personalized approach to immune checkpoint inhibitors.

In another study aiming to predict which anaplastic thyroid carcinoma patients may benefit from immunotherapy, the study by Pengping et al. revealed that HLF gene hinders cancer progression by down-regulating the epithelial-to-mesenchymal transition pathway, reducing T cell exhaustion, and increasing sensitivity to sorafenib kinase inhibitor.

Finally, Wellhausen et al. showed how pembrolizumab, a PD-1 inhibitor, can enhance the efficacy of neoadjuvant chemotherapy in *in vitro* studies. This study reported that suppression of MCP-1, IFN-γ and IL-6 production by pembrolizumab in head and neck squamous cell carcinoma was linked to improved survival. This work highlights the synergistic effects of immune checkpoint inhibitors and chemotherapy, which are mediated by the inhibition of inflammatory cytokines.

